# A SURVEY OF MICROGLIA ASSOCIATION WITH AMYLOID PLAQUE IN 3XTG ALZHEIMER’S DISEASE MODELS

**DOI:** 10.1093/geroni/igae098.3628

**Published:** 2024-12-31

**Authors:** Anthony Asante-Danso, Mofida Abdelmageed, Anirban Paul

**Affiliations:** Howard University, Washington, District of Columbia, United States; Penn State College of Medicine, Hershey, Pennsylvania, United States; Penn State College of Medicine, Hershey, Pennsylvania, United States

## Abstract

Brain aging is characterized by a progressive functional decline of cellular and molecular mechanisms that affect most living organisms. These mechanisms’ decline is the primary risk factor for many neurodegenerative diseases, including Alzheimer’s Disease (AD). In neurodegenerative diseases like AD, extracellular deposition of amyloid-beta (Aβ) and microglial activation are critical facets that influence disease progression. Microglia, the resident immune cells of the central nervous system, play a key role in maintaining brain homeostasis and eliminating these Aβ forms through phagocytosis. However, increased microglia association with amyloid plaque can lead to neuroinflammation, thus creating a more neurotoxic environment in the brain, leading to further AD progression and neuronal loss. Little research examines how this association of microglia and amyloid plaque differs with sex or age. To address research gaps, we are using wild-type and triple transgenic human-induced AD models (3xTg-AD) of female and male mice to gain a strong understanding of the changes in microglia activation in brain aging and AD.

